# Enhanced CD56 Expression and Increased Number of CD56^+bright^ Cells in the Peripheral Blood of Untreated Endometriosis Patients

**DOI:** 10.1111/aji.70214

**Published:** 2026-02-04

**Authors:** Emma Björk, Pernilla Israelsson, Olga Nagaeva, Lucia Mincheva‐Nilsson, Ulrika Ottander

**Affiliations:** ^1^ Division of Obstetrics and Gynecology/Örnsköldsvik Hospital Örnsköldsvik Sweden; ^2^ Department of Clinical Microbiology/Infection and Immunology Umeå University Umea Sweden; ^3^ Department of Clinical Sciences/Obstetrics and Gynecology Umeå University Umea Sweden; ^4^ Department of Diagnostics and Intervention/Oncology Umeå University Umea Sweden

**Keywords:** CD56^+bright^, endometriosis, immunosuppressive exosomes, NK cells, NKG2D

## Abstract

**Problem:**

NK‐cell dysfunction in endometriosis is suggested to contribute to the survival of ectopic endometrial tissue. However, the underlying causes of this impairment remain unclear. NK cells are divided into: CD56^+bright^, which produce high amounts of cytokines but have low or no cytotoxic ability, and CD56^+dim^, which are mainly cytotoxic. CD56^+bright^ NK cells, constitutively present in human endometrium (eNK cells), represent only 0–2% of NK cells in PBMC, where CD56^+dim^ cells dominate.

**Method of Study:**

NK‐cell subpopulations and NKG2D receptor expression in PBMC were analyzed by flow cytometry in two cohorts of untreated and treated endometriosis patients and healthy age‐matched controls.

**Results:**

Elevated numbers of CD56^+bright^ cells were observed in 8 of 21 untreated endometriosis patients compared to controls. These numbers were normalized following surgery and hormonal treatment. The NKG2D receptor expression was reduced in untreated patients compared to controls and treated patients.

**Conclusions:**

The significantly increased proportion of peripheral CD56^+bright ^NK/eNK cells may result from migration of these cells from ectopic endometrial tissue. The downregulation of NKG2D receptor expression in PBMCs may be mediated by immunosuppressive endometriotic exosomes, as previously reported by us. Taken together, our results suggest that: (1) the impaired NK cell cytotoxicity in untreated endometriosis patients may be due both to an influx of CD56^+bright^/eNK cells and exosome‐induced NKG2D receptor downregulation; and (2) elevated numbers of peripheral CD56^+bright^ NK cells could be considered as a potential diagnostic marker for endometriosis.

## 1 Introduction

Endometriosis is a chronic disease affecting approximately 10% of women of reproductive age. It is characterized by ectopic growth of endometrial‐like tissue, leading to symptoms such as dysmenorrhea, pain, infertility, and an increased risk of certain types of cancer [1, 2]. Despite extensive research, the underlying molecular and cellular mechanisms of the disease remain unclear. The most widely accepted explanation for the ectopic spread of endometrial tissue is retrograde menstruation [3, 4]. However, nearly all women have retrograde menstruation, and additional factors, including genetic predisposition, chronic inflammation, and immune dysfunction, have been implicated in the complex pathogenesis of endometriosis.

Emerging evidence suggests a dysregulation of key immunological processes in endometriosis, supporting the novel view of the disease as a multifactorial, immune‐mediated disorder. Impaired immune responses are reported to allow ectopic endometrial tissue to evade immunosurveillance, resulting in inadequate detection and clearance of these cells [5, 6, 7]. Natural killer (NK) cells, cells of the innate immune defense, are expected to eliminate endometrial cells displaced by retrograde menstruation. However, increasing evidence suggests that NK cells in endometriosis patients exhibit compromised cytotoxic function, failing to eliminate ectopic endometrial tissue, thereby contribute to disease development and progression. A suggested mechanism for the NK cell dysfunction is impaired cytotoxicity due to downregulation of the NKG2D receptor and altered NK‐cell receptor‐ligand interactions caused by immunosuppressive exosomes. Additionally, aberrant cytokine responses such as elevated levels of immunosuppressive cytokines (TGF‐β, IL‐10, and IL‐6), together with reduced production of IFN‐γ are thought to contribute to the immune dysfunction. Together, these factors contribute to the compromised cytotoxicity of NK cells observed in endometriosis [8, 9, 10, 11, 12, 13].

In this study, we investigated CD56^+^NK cells in peripheral blood samples from two cohorts of women with severe endometriosis (rASRM stage III and IV). Using immunofluorescent staining and flow cytometry, we analyzed peripheral blood lymphocytes (PBMC) with a focus on the two NK‐cell subtypes, CD56^+dim^ and CD56^+bright^. We found that a group of endometriosis patients exhibited elevated numbers of CD56^+bright^ NK cells compared to healthy age‐matched controls. To investigate these findings further, we analyzed the number and distribution of CD56^+bright^ NK cells in these patients before and after treatment, comparing them with those in healthy age‐matched controls. Additionally, we analyzed NKG2D receptor expression on the NK‐cell subsets and cytotoxic CD8^+^ T cells before and after treatment, and compared the results with those of healthy controls.

## 2 Materials and Methods

### 2.1 Collection of Samples and Study Population

**FIGURE 1:**
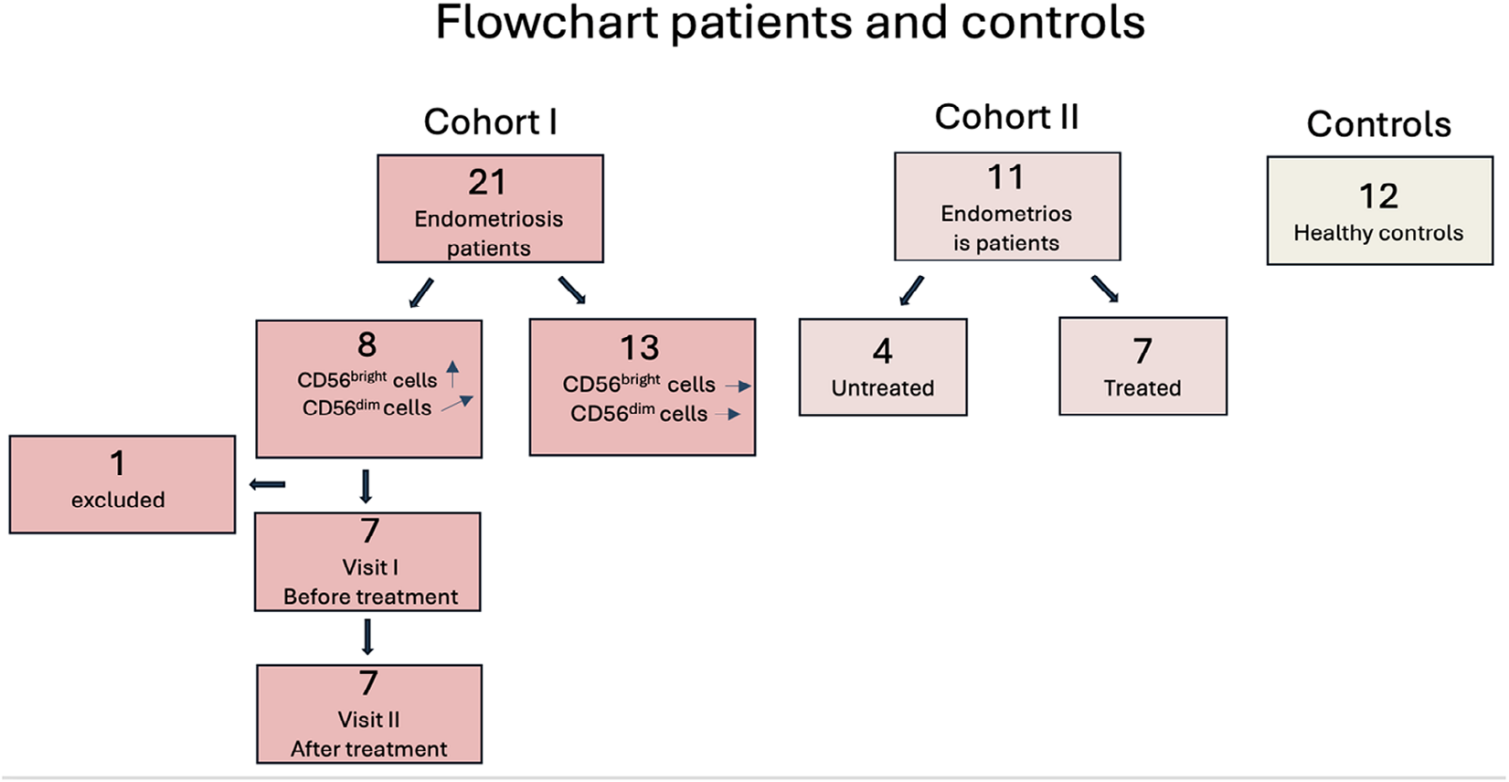
Flowchart illustrating the two cohorts and controls.

Peripheral blood samples were collected from 21 endometriosis patients at the Women's Clinics at Norrland's University Hospital (Umeå) and Örnsköldsvik Hospital, following ethical approval by the Human Ethics Committee of the Medical Faculty, Umeå University (d.nr 09–108M) and written informed consent. An initial finding of elevated CD56^+bright^ NK cell numbers in 8 of these patients prompted us to study this further. Follow‐up samples were obtained from all but one patient (who had relocated) after surgery and hormonal treatment. These patients constituted Cohort I (Figure 1). Additionally, blood samples were collected from a second group of 11 endometriosis patients (four untreated and seven treated), constituting Cohort II (Figure 1). All patients were of fertile age and diagnosed with stage III and IV endometriosis according to the rASRM classification [14]. A pathologist verified the histopathological diagnoses. Serum samples from age‐matched healthy controls (*n =* 12) were also included for comparison (Figure 1). The sample collection was not synchronized with the menstrual cycle.

### 2.2 Antibodies Used in this Study

The following antibodies were used: CD45/CD14 Leucogate (BD Biosciences); CD4/RPE (Agilent DAKO); CD8/FITC (Agilent DAKO); CD16/FITC (Agilent DAKO); CD19/RPE (Agilent DAKO); CD56/Alexa Fluor 647 (BD Pharmingen); NKG2D/ RPE (BD Pharmingen); CD161/RPE (BioLegend).

### 2.3 Isolation of PBMC From Patients and Healthy Donors

PBMC were isolated from the peripheral blood of endometriosis patients and healthy controls within 24 h of obtaining the samples. In brief, peripheral blood samples were collected in heparin‐treated tubes and kept at 4°C during transportation to the lab. The blood was diluted with an equal number of Tris‐Hanks solution (pH 7.5) and distributed in tubes with Ficoll‐Isopaque (Lymphoprep, Nycomed) solution by carefully overlaying it on the top of the solution. Gradient centrifugation for 30 min at 2000 rpm without a break was performed, and the interphase containing PBMC was collected. After 3 x washing with Tris‐Hanks solution, the cells were resuspended in HEPES‐buffered RPMI (Gibco) supplemented with 8% FCS and antibiotics to a concentration of 10^6^/ml and used in immunofluorescence staining with mAbs for phenotype and receptor markers. By omitting a freezing step after PBMC isolation, cell death due to freezing and thawing is avoided, allowing the preservation of activated or cells otherwise sensitive to freezing and thawing.

### 2.4 Immunofluorescence Staining of PBMC

Phenotypic and activation markers on PBMCs from patients and controls were analyzed using flow cytometry with mAbs and immunofluorescence staining. In brief, one hundred thousand cells/well were plated on U‐shaped microtiter plates in PBS containing 3% bovine calf serum and 0.05% sodium azide. Immunofluorescence staining with directly labeled mAbs with FITC‐ and/or PE was used for most phenotypic markers. Appropriately labeled, isotype‐matched irrelevant mAbs (DAKO) were used in the negative controls. As secondary antibodies FITC‐conjugated goat anti‐mouse antibodies from DACO were used. Double immunofluorescent stainings were performed using mAbs against CD56/NKG2D and CD8/NKG2D. A minimum of 10 000 events for each marker staining were collected using an Accuri C6 flow cytometer (BD Biosciences) and analyzed with CFlow Plus software (BD Biosciences).

### 2.5 Statistical Analyses

The GraphPad Prism program was used in the statistical analyses. Comparison between groups was calculated with a two‐sided Student's *t*‐test and *p* ≤ 0.05 was considered significant.

## 3 Results

### 3.1 Patients and Controls

Patient and control characteristics are summarized in Table 1. Of the initial 21 serum samples collected from endometriosis patients, eight were selected for further study based on their peripheral blood NK cell profile (Figure 1) (referred to as Cohort I). At the time of the first blood sample (Visit I), five of these eight patients were not receiving hormonal treatment, one had a hormonal intrauterine device (IUD), and one had both an IUD and systemic low‐dose medroxyprogesterone acetate (MPA), 5 mg/day. Following surgical and hormonal (progestogen) treatment, new blood samples were collected from all eight patients at Visit II, except one who had relocated. To further explore our hypothesis, an additional cohort of 11 endometriosis patients (Cohort II) was included, and blood samples were collected from four untreated and seven patients treated by surgery and subsequent progestogen (Table 1, Figure 1). In all patients undergoing surgery, all visible endometriotic tissue was removed.

**TABLE 1:** Characteristics of endometriosis patients and controls.

	Cohort I				
		*Visit I*	*Visit II*	Cohort II	Controls
*Patients*	21	7	7	11	12
*Serum samples*	21	7	7	11	12
*Age*					
Mean	35,7	36,1		38,5	40,2
Max	47	47		48	52
Min	22	24		33	19
*Parity*					
0	13	4		3	1
1	3	1		2	1
2	4	1		5	8
3 or more	1	1		1	2
*Treatment*					
None	17	5	—	3	12
IUD	3	1	—	1	—
IUD + MPA	1	1	—	—	—
Surgery + progestogen	—	—	7	7^a^	—
*Anti‐inflammatory drugs* ^b^					
Yes	—	—	—	—	—
No	21	7	7	11	12
*Dysmenorrhea*					
None	3	—		2	7
Mild	—	—		—	3
Moderate	7	4		3	2
Severe	11	3		6	—
*Histopathology verified endometriosis*					
Yes	21	7		11	NA
No	—	—		—	NA
*Stage according to rASRM*					
III	9	4		6	NA
IV	12	3		5	NA

Abbreviation: IUD =  intrauterine device, levonorgestrel 20 µg/24h, MPA = medroxyprogesterone acetate 5 mg/day, NA = not applicable.

^a^ Designated as “treated” in Cohort II.

^b^ COX‐inhibitors or glucocorticoids.

### 3.2 Phenotypic Analyses of the PBMCs From Endometriosis Patients and Controls Show an Equal Distribution of Lymphocyte Subsets

**FIGURE 2:**
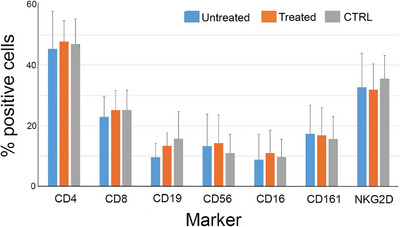
Phenotype of lymphocytes in endometriosis patients from Cohort I and II, and controls; untreated patients (*n =* 25), treated patients (*n =* 14), and controls (*n =* 12).

We assessed the lymphocyte phenotypes in the peripheral blood from untreated (*n =* 25; 21 from Cohort I and 4 from Cohort II) and treated (*n =* 14; 7 from each cohort) endometriosis patients, and from controls (*n =* 12), using immunofluorescence staining of phenotypic markers and flow cytometry (Figure 2). There were no statistically significant differences in the percentage of helper (CD4^+^) and cytotoxic (CD8^+^) T cells, B cells (CD19^+^), NK cells (CD56^+^), CD16/Fc γ receptor‐expressing cells or the cells expressing the *c*‐type lectin superfamily receptors CD161/KLBR1 and NKG2D among untreated or treated endometriosis patients and controls.

### 3.3 Increased Numbers and Enhanced CD56 Expression of Circulating CD56^+bright—^And CD56^+dim^ NK Cells in a Group of Untreated Endometriosis Patients Decreased After Treatment

**FIGURE 3:**
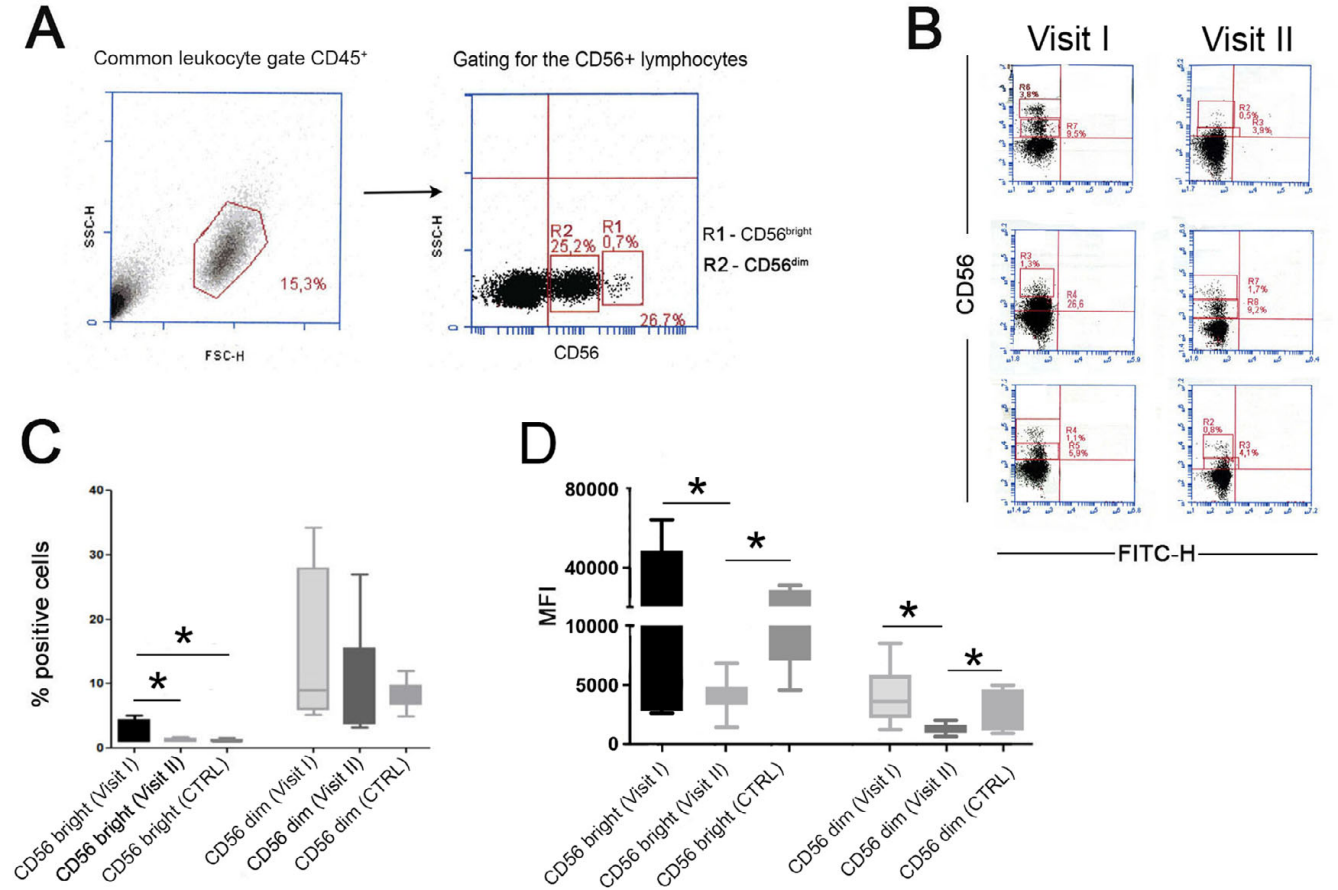
CD56^+bright^ and CD56^+dim^ NK cell expression in peripheral blood of endometriosis patients (Cohort I, *n =* 7), and healthy controls (*n =* 12). (A) Gating strategy. (B) Representative dot blots of flow cytometry experiments showing the distribution of CD56^+bright^ and CD56^+dim^ NK cells in endometriosis patients at Visit I (pre‐treatment) and II (post‐treatment). (C) Summary of the percentage of CD56^+^ bright and dim NK cells in endometriosis patients compared to. controls. CD56^+bright^ cells were significantly elevated in untreated patients (Visit I) and normalized after treatment (Visit II) (Visit I vs. Visit II: *p =* 0.02; Visit I vs. controls (*p =* 0.009). (d) Mean fluorescent intensity (MFI) of CD56^+bright^ and CD56^+dim^ NK cells. CD56^+bright^ MFI decreased significantly after treatment (Visit I vs. II: *p =* 0.05) and was lower at visit II compared to controls (*p =* 0.006). CD56^+dim^ MFI was also significantly reduced at Visit II compared to both Visit I (*p =* 0.002) and controls (*p =* 0.04.

We analyzed the bright and dim NK cell subsets in peripheral blood from endometriosis patients in Cohort I, collected at Visit I (before treatment) and Visit II (after treatment), and compared them to healthy controls. The results are summarized in Figure 3. In seven patient samples at Visit I, we observed an increased proportion of CD56^+bright^ cells compared to both Visit II controls. This is illustrated in Figure 3b, which shows representative dot plots from three patients. Figure 3c summarizes the CD56^+bright^ and CD56^+dim^ subset analysis across all seven patients from Cohort I. The percentage of CD56^+bright^ cells was significantly higher at Visit I than in controls (*p =* 0.009, Figure 3c) and decreased significantly after treatment (*p =* 0.02). Although the percentage of CD56^+dim^ NK cells also declined after treatment (Visit II), closer to that of controls, this change was not statistically significant (Figure 3c). The density of CD56 expression, assessed by MFI, showed that the intensity of CD56^+bright^ cells was significantly higher before treatment (Visit I) compared to after treatment (Visit II) (*p =* 0.05, Figure 3d). The CD56^+dim^ expression was also significantly lower at Visit II compared to both Visit I (*p =* 0.002, Figure 3d) and controls.

### 3.4 Differential Response to Treatment With Normalized NKG2D Expression on CD56^+^ but Not on CD8^+^ PBMCs From Endometriosis Patients

**FIGURE 4:**
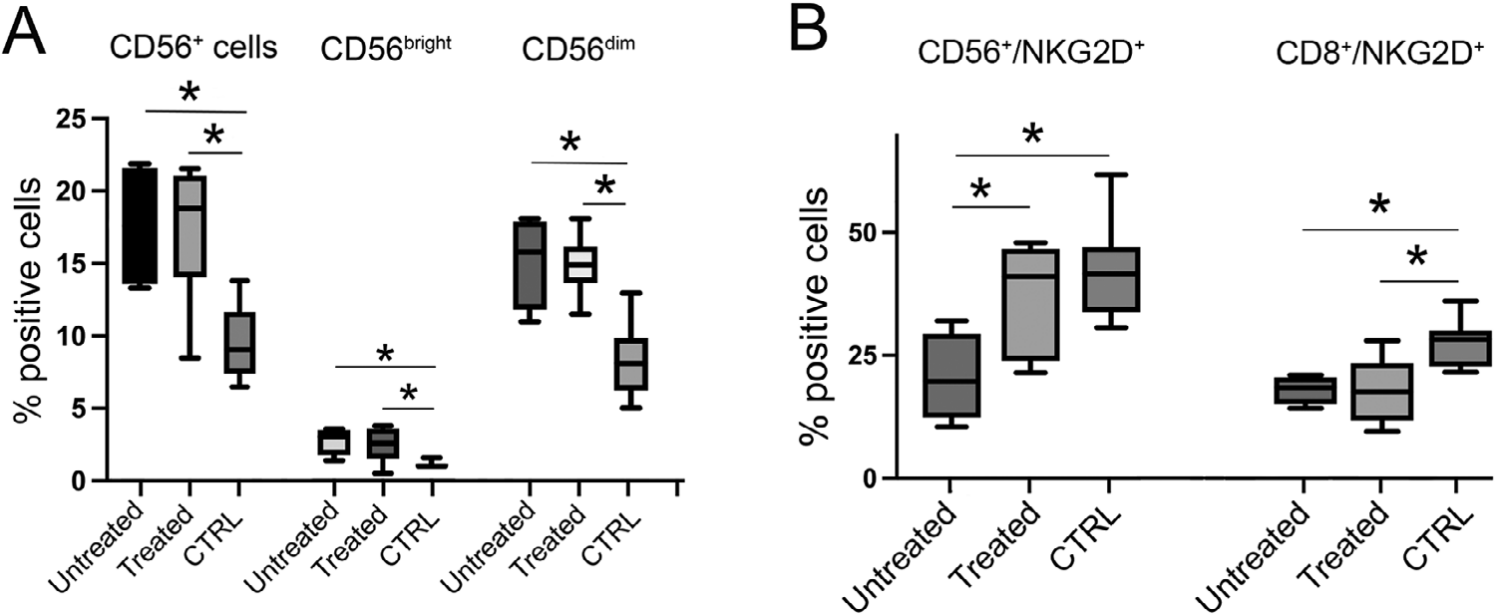
Assessment of PBMC expressing CD56, CD8, and NKG2D, using flow cytometry and gating on CD56^+^ cells (A) and CD45^+^ cells (B) in endometriosis patients of Cohort II, untreated (*n =* 4), treated (*n =* 7) and controls (*n =* 12). (1) CD56^+^ cells untreated vs. controls (*p =* 0.02), treated vs controls (*p =* 0.004) (A); (2) CD56^+bright^ cells untreated vs. controls (*p =* 0.04), treated vs. controls (*p =* 0.03) (A); (3) CD56^dim^ cells untreated vs. controls (0.014), and treated vs. controls (*p =* 0.0002) (A); (4) NKG2D^+^ CD56^+^ cells untreated vs. treated (*p =* 0.04), and untreated vs. controls (*p =* 0.01) (b); 5) NKG2D^+^ CD8^+^ untreated vs. controls (*p =* 0.001), treated vs. controls (*p =* 0.005) (B).

Next, we studied NKG2D receptor expression in PBMCs from untreated (*n =* 4) and treated (*n =* 7) endometriosis patients in Cohort II, compared to healthy controls (*n =* 12). A similar experiment as in Cohort I was used to assess the percentage of CD56^+^ cells and CD56^+^ subsets. As shown in Figure 4a, both untreated and treated patients exhibited significantly higher percentages of CD56^+^ cells compared to controls (*p =* 0.02 and 0.004, respectively), with a somewhat lower expression in treated patients relative to untreated. The same can be seen in the percentage of CD56^+bright^ and CD56^+dim^ cells. While there is no significant difference between the treated and untreated patients, both groups displayed elevated levels compared to controls: CD56^+bright^ cells in untreated vs. controls (*p =* 0.04), treated vs. controls (*p =* 0.03) and CD56^+dim^ cells in untreated vs. controls (*p =* 0.014) and treated vs. controls (*p =* 0.0002). PBMCs were double‐stained and gated for CD56 and NKG2D, as well as CD8 and NKG2D (Figure 4b). Interestingly, the NKG2D expression on CD56^+^ cells was significantly elevated in treated compared to untreated patients (*p =* 0.04). Expression levels in controls was comparable to those of treated patients, and thus also significantly higher than in untreated patients (*p =* 0.01). In contrast, the percentage of CD8^+^ NKG2D^+^ cells remained unchanged between treated and untreated groups and was slightly lower than in controls (Figure 4b).

## 4 Discussion

In this study, we investigated the phenotype of NK cells, specifically CD56‐ and NKG2D receptor expression, in the peripheral blood of endometriosis patients before and after treatment and compared the findings to healthy controls. The main findings are summarized as follows: (1) there was no statistically significant difference in total CD56^+^ NK cell percentage in untreated, treated patients, and controls, consistent with previous reports. (2) However, there were qualitative differences in the CD56^+^ expression levels. Untreated endometriosis patients showed significantly enhanced numbers of CD56^+bright^ NK cells, which normalized after treatment. This may reflect a redistribution of CD56^+bright^ NK cells from ectopic endometrial lesions to the peripheral blood. (3) NKG2D expression on NK cells was significantly reduced in untreated patients, but restored after treatment to the levels of healthy controls. A possible reason for this recovery may be the surgical removal of ectopic endometrial tissue, which has been shown to release immunosuppressive NKG2D ligand‐carrying exosomes [11]. Taken together, our results suggest that the impaired NK‐cell cytotoxicity observed in endometriosis patients may in part result from the systemic presence of CD56^+bright^ NK cells, which are less cytotoxic, originating from endometriotic lesions. Additionally, blockage of NKG2D receptors by ligand‐bearing exosomes secreted by endometriotic lesions may contribute to the observed downregulation of the NKG2D receptor in the peripheral blood of untreated endometriosis patients.

As mentioned, no statistical significance in overall lymphocyte percentages was observed when phenotyping PBMCs from untreated and treated patients and controls. However, we observed that 8 out of 21 untreated patients in Cohort I had significantly elevated levels of CD56^+bright^ NK cells, as illustrated in the dot plot data. The CD56^+dim^ NK cells were also increased compared to healthy controls, though not significantly. Moreover, CD56 expression intensity was higher in CD56^+bright^ NK cells from untreated patients than that of the healthy controls, suggesting increased activation. Follow‐up samples were analyzed to assess whether treatment normalized these alterations. Notably, the elevated levels of CD56^+bright^ NK cells observed prior to surgery declined to control levels after surgical and hormonal treatment. This finding was confirmed in Cohort II. Consistent with our results, increased peripheral CD56^+bright^ NK cells in endometriosis patients compared to controls were recently reported by [15]. Human NK cells can be divided into CD56^+dim^ and CD56^+bright^ subsets, based on CD56 expression levels. Most “conventional, mature” circulating human NK cells are denominated as CD56^+dim^CD16^+^, characterized by high cytotoxic ability—natural, receptor‐mediated cytotoxicity and antibody‐dependent cell‐mediated cytotoxicity (ADCC) mediated by CD16. In contrast, CD56^+bright^ NK cells are more common in tissues, lymph nodes, and tonsils, and scarce in peripheral blood. They are characterized by high production of immunosuppressive cytokines such as TGFβ and IL‐10, but minimal cytotoxicity [16, 17]. Both NK cell subsets express the major activating NK‐cell receptor NKG2D, which is crucially important for immune surveillance [18].

In healthy individuals, CD56^+bright^ NK cells are rare in peripheral blood, comprising only 0%–2% of PBMCs. A distinct subset of CD56^+bright^ CD16^−^ NK cells, termed uterine NK (uNK) cells, permanently resides within the uterine mucosa. These include endometrial (eNK) and decidual (dNK) NK cells, which account for 30% of endometrial lymphocytes and expand to 50%–70% as dNK cells in the decidua during the first trimester of pregnancy [16, 17]. It is plausible that eNK cells, shed with the endometrial tissue during retrograde menstruation, persist in the implanted endometriotic lesions and, from there, enter the peripheral blood. Fukui et al. demonstrated that the predominant NK cell population in peritoneal fluid from endometriosis patients consists of CD56^+bright^ NK cells that is, eNK cells. [18]. We suggest that these eNK cells enter the peripheral blood, where their low cytotoxic capacity and suppressive/regulatory cytokine profile [19] impair the overall cytotoxic ability in endometriosis. Our investigation of the cytokine mRNA profiles in endometriosis [20] supports this hypothesis. The amount of CD56^+bright^ NK cells in peripheral blood is expected to be considerably lower than in peritoneal fluid. Nevertheless, elevated levels were observed in 8 out of 21 patients, although the exact relationship remains unknown. Albeit in small cohorts, our data show that significantly elevated peripheral CD56^+bright^ NK cell/eNK cell levels might underlie the observed dysfunction/cytotoxic incapability of NK cells in endometriosis. This is supported by our results showing that, following surgery and/or hormonal treatment, the numbers of CD56^+bright^ NK cells in the peripheral blood of endometriosis patients drop to the levels of healthy controls, suggesting that the treatment prevents their leakage into the circulation. In line with our results, a previous study reports that surgery increases the number of mature “conventional” peripheral CD56^+^/CD16^+^ NK cells [21].

The next step in our study was to investigate the expression of the NKG2D receptor on peripheral NK cells and CTLs in Cohort II patients. In untreated patients, CD56^+^ NK cells displayed significantly lower NKG2D receptor expression compared to healthy controls. By contrast, treated patients showed expression levels similar to those of the control group. However, there was no significant difference in NKG2D expression on CD8^+^ NK cells observed between untreated and treated patients. A possible explanation for the reduced NKG2D receptor levels in untreated patients is downregulation induced by exosomes carrying NKG2D ligands. In a recent report, we demonstrated that short cultures of ectopic endometrial explants secrete exosomes that carry NKG2D ligands on their surface, which are able to downregulate the NKG2D receptor and impair the cytotoxic ability of NK cells, a mechanism that ensures survival and growth of endometriotic lesions. [11]. Our results are supported by a report [22] showing elevated levels of soluble NKG2D ligands—likely exosome‐associated—in the peritoneal fluid of endometriosis patients, which could impair NKG2D‐mediated cytotoxicity. In contrast, Xu et al. reported reduced NKG2D expression on NK cells isolated from the peritoneal fluid of endometriosis patients, but not in peripheral blood [23]. Dysregulation of CD8^+^ T cells has previously been studied in endometriosis, where it was shown that they lack the fluctuating levels observed in healthy controls throughout the menstrual cycle [24]. However, NKG2D expression on CD8^+^ T cells has not previously been reported in endometriosis, and the divergent treatment responses between CD56^+^ NK cells and CD8^+^ T cells observed in our study could be an interesting subject for further investigation. Our cohort includes ovarian endometriomas, which are considered a type of deep infiltrating endometriosis, and only rASRM stage III and IV. It would be of interest to study the relationship between all subtypes and stages of endometriosis and NK cells.

In summary, we found that untreated endometriosis patients generally had NK cells with higher CD56 expression intensity levels, and some showed elevated numbers of CD56^+bright^ NK cells in the peripheral blood, likely due to leakage from endometriotic lesions. These levels normalized following radical surgery and subsequent progestogen treatment. We also demonstrated lower NKG2D expression on both dim and bright CD56^+^ NK cells in patients compared to controls, likely due to immunosuppressive exosomes released by endometriotic tissue. NKG2D levels returned to normal after surgical removal of endometriotic tissue. The main disadvantage of this study is the small sample size, mainly due to challenges in recruiting untreated endometriosis patients. In Sweden, most patients with dysmenorrhea receive hormonal treatment in line with national guidelines. Additionally, Northern Sweden, the catchment area for this study, is sparsely populated. Thus, these observations should be viewed as pilot or proof‐of‐concept studies. Despite this, our findings might be of interest for (1) evaluating peripheral CD56^+bright^ NK cells as potential diagnostic markers for progression or regression of endometriosis, as previously shown in malignant melanoma [25], and (2) assessing responses to endometriosis treatment. Future studies involving larger patient cohorts and defined subgroups of patients, stratified by disease severity and treatment types, should be investigated. A thorough analysis regarding clinical, pathological, hormonal, and immunological characteristics in relation to the CD56^+^NK cell subset should be made. It would also be of interest to perform functional cytotoxicity experiments with peripheral CD56^+bright^ and CD56^+dim^ NK cells of endometriosis patients before and after treatment. These aspects need to be explored in further studies. There are similarities between endometriotic and malignant tissues, such as invasion, angiogenesis, and inflammation [26]. Thus, our results suggest that NK cell‐based therapies that are currently under investigation in oncology should be explored in endometriosis [27, 28, 29, 30, 31].

## Ethics Statement

Ethical approval for this study was obtained from the Human Ethics Committee of the Medical Faculty, Umeå University (d.nr 09–108M). Samples were collected after written informed consent.

## Conflicts of Interest

All authors declare that they have no conflicts of interest.
